# Association Between Breast Microbiota and Capsular Contracture: A Systematic Review

**DOI:** 10.1093/asjof/ojaf128

**Published:** 2025-10-07

**Authors:** Hassan Aden, Abdirahman Ahmed Mohamud, Abdisalam Ismail Hassan, Osman Abubakar Fiidow, Ahmed Muhammad Bashir

## Abstract

Capsular contracture (CC), a common complication of breast implants, has an unclear etiology. Subclinical infection around the implant is widely considered a potential cause. Microorganisms, such as *Staphylococcus epidermidis*, have been associated with CC, but the specific role of bacteria from the breast capsule, glandular tissue, or skin remains unclear. No molecular studies have definitively investigated this association. The authors of this systematic review aim to evaluate the relationship between breast microbiota, bacterial biofilms, and CC in patients undergoing cosmetic or reconstructive breast augmentation. Data were extracted from studies identified through different search engines, including Medline and Embase. Inclusion criteria focused on patient, surgical, and implant-related factors influencing CC. Only English-language articles were considered. The review included 428 women (453 breast implants) aged 27 to 53 years, with an average age of 31. Most studies lacked detailed reporting on implant characteristics or surgical techniques. The majority employed cell culture or pathology for microbiota assessment, with 1 study using polymerase chain reaction (PCR). Frequently identified bacteria included *S. epidermidis*, *Propionibacterium acnes*, and *Streptococcus* spp. This systematic review of 428 women with 453 breast implants found that *S. epidermidis*, *P. acnes*, and *Streptococcus* spp. were the most frequently isolated microorganisms in CC cases. The majority of studies used cell culture or pathology for microbiota assessment, with 1 employing PCR. Bacterial biofilm, particularly involving *S. epidermidis*, was consistently reported in contracted capsules, suggesting a strong association between specific breast microbiota and CC.

**Level of Evidence: 2 (Risk)**  
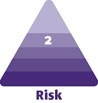

Breast augmentation and reconstruction are among the most common surgical procedures worldwide, performed for both aesthetic enhancement and postmastectomy reconstruction. Despite their prevalence, complications such as capsular contracture (CC) pose significant challenges, affecting patient satisfaction and surgical outcomes. CC is characterized by an abnormal fibrotic response around the breast implant, leading to discomfort, pain, and aesthetic deformities.^1^ Although its exact etiology remains unclear, emerging evidence suggests a strong link between bacterial biofilm formation and the onset of CC.^2^

The role of breast microbiota in implant-associated complications has gained increasing attention in recent years. Biofilms—structured bacterial communities encased in an extracellular matrix—are known to colonize implant surfaces, triggering chronic inflammation and fibrosis.^3^ Studies have identified *Staphylococcus epidermidis*, *Propionibacterium acnes*, and *Streptococcus* spp. as the most frequently isolated microorganisms in contracted breast capsules.^4^ These bacteria, commonly part of the skin microbiome, may infiltrate the implant pocket during surgery, subsequently forming biofilms that contribute to CC development.^5^

Although bacterial biofilms have been implicated in CC pathogenesis, their exact contribution remains a subject of ongoing investigation. Factors such as implant texture, surgical techniques, and host immune responses may further modulate the risk of biofilm formation and subsequent fibrosis.^6^ In addition, the breast implant-associated anaplastic large cell lymphoma (BIA-ALCL) has been linked to microbial dysbiosis, suggesting that implant-associated infections may have broader clinical implications.^7^

This review is specifically intended to synthesize the current evidence regarding the microbial species identified in pathologic breast implant capsules and their association with CC. Although related conditions and prevention strategies are briefly noted for context, the focus remains on cataloging and interpreting the microorganisms reported in the literature.

## METHODS

### Search Strategy

The analysis in this study is based on data collected from multiple databases, specifically Medline and Embase. The search strategy utilized a combination of keywords, including “female breast,” “breast augmentation,” “breast reconstruction,” “mammaplasty,” “breast implant,” “breast prosthesis,” “postoperative complication,” “capsular contracture,” “microbiota,” “bacteria*,” and “microbe*.” Boolean operators (“AND” and “OR”) were applied to refine the search and ensure comprehensive coverage of relevant literature.

Both Medline and Embase, along with references cited in the included literature, were systematically searched. The study was limited to peer-reviewed publications available in English. No temporal restrictions were applied during the search phase, allowing the inclusion of studies regardless of publication date.

This systematic review followed the Preferred Reporting Items for Systematic Reviews and Meta-Analyses (PRISMA) guidelines to ensure transparency and methodological rigor. However, the review was not prospectively registered in an international database such as PROSPERO or INPLASY. The absence of prospective registration is acknowledged as a limitation, although all steps of protocol development, study selection, data extraction, and analysis were conducted in accordance with PRISMA standards.

### Method of Criteria Selection

We sought original research investigating the microbiome and its association with CC. Articles were included if they addressed CC as a consequence of breast implantation, either for cosmetic breast augmentation or breast reconstruction in breast cancer patients. The focus was on patient-related, surgical, and/or implant-related factors. Abstracts, case reports, reviews, editorials, communications, correspondence, dialogues, and letters were excluded. Only studies published in English were considered.

Publications were screened based on predefined inclusion and exclusion criteria by reviewing titles and abstracts. If the title and abstract were ambiguous, the full article was examined. Additional cross-referencing was conducted using the UCL Medical Library, Royal Free Medical Library, and Ovid Web of Science to identify and verify relevant studies. Full texts of selected articles were thoroughly evaluated to ensure they met the eligibility criteria.

### Inclusion–Exclusion Criteria

This systematic review employed the PICO framework (Population, Intervention, Control, and Outcomes) to establish inclusion and exclusion criteria. The inclusion criteria focused on human studies, specifically involving females who underwent breast implant surgery for aesthetic or reconstructive purposes and subsequently developed CC. Additionally, studies with laboratory-based results investigating breast microbiota and bacterial involvement were considered eligible.

Exclusion criteria eliminated studies involving nonhuman participants (eg, animal studies), male breast procedures, and breast surgeries without implants, because these were not directly relevant to CC. Clinical reports lacking microbiological investigation were also excluded to ensure data quality and relevance (Table 1).

**Table 1.** Inclusion and Exclusion Criteria

Inclusion criteria	Exclusion criteria
Human study	Animal study
Female	Male
Breast implant surgery for aesthetic and reconstructive reason	Breast augmentation/reconstruction without implants
Presenting capsular contracture	Another complication other than capsular contracture
Lab-based assessment of biofilm/microbiota/microorganisms in general/bacteria	Clinical reports without microbial analysis or microbial involvement or investigation

To ensure comprehensive literature inclusion, selected articles needed to explicitly address the relationship between breast microbiota and CC. Only studies presenting data from human research, particularly focusing on female breast implant surgeries, were analyzed. This narrowed focus inherently included some articles that were less relevant but were necessary to avoid excessive keyword refinement, which risked omitting valuable data and potentially misrepresenting the CC phenomenon.

The systematic approach carefully evaluated each item against the inclusion and exclusion criteria, ensuring that only appropriate studies were retained. This rigorous process mitigated bias and maintained the scientific integrity of the review while discarding irrelevant articles.

### Collection and Analyzes of Data

The incidence and severity of CC were collected from the included papers, as were the study, patient characteristics, the indication for surgery, type of operation, implant characteristics, and microbiome data. The factors listed in Table 2 were included in the data.

**Table 2.** Factors Included in Data

Category	Variable
Features of the research	Author(s) Year of publication Country Title Sample size Sample size (number of patients) Sample size (number of the breast)
Characteristics of patients	Age The number of women (for an aesthetic reason) surgery Number of women (for a reconstructive reason) surgery If reconstructive, which type of cancer chemo/radiotherapy
Implant characteristics	Implant size Implant surface (textured or smooth) Implant shape (round or anatomical) Type of implant (silicone or saline) Position of implant (subglandular or submuscular)
Microbiome characteristics	Methods of assessing the specimen (type of lab test on pathogens) Species of bacteria found Incidence of bacteria (number of patients)
Capsular contracture characteristics	Incidence of capsular contracture (number of breast) Severity of capsular contracture Baker classification score (which grade) Incidence of BIA-ALCS

BIA-ALCS, breast implant-associated anaplastic large cell lymphoma.

## RESULTS

The Medline database yielded a score of 25, whereas the Embase database yielded a score of 48.

**Figure 1.**
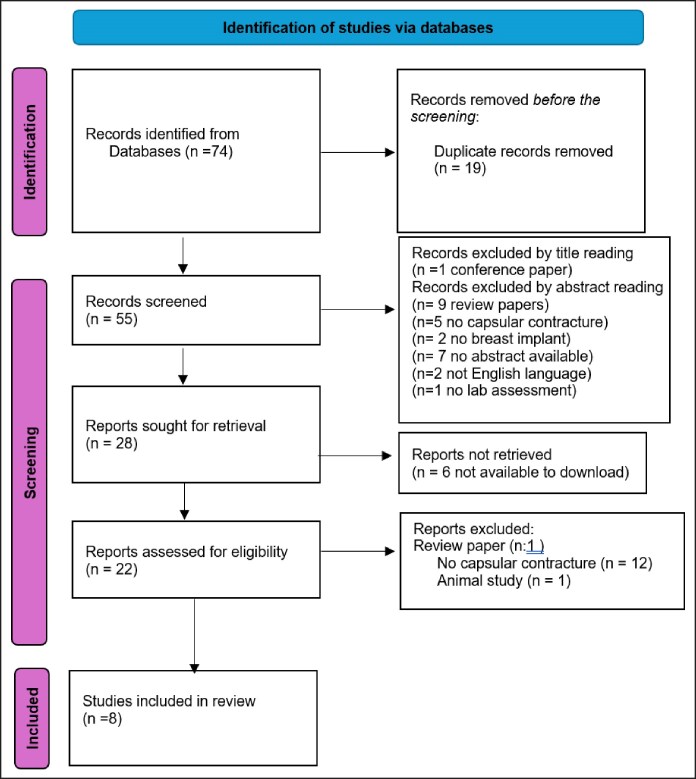
The blow Preferred Reporting Items for Systematic Reviews and Meta-Analyses chart flow diagram describes the process of doing a literature search.

The PRISMA flowchart indicates that 74 articles were selected from primary and secondary sources, including 2 major databases, Medline and Embase (Figure 1).

The factors stated in the inclusion and exclusion criteria were used to guide the selection of publications. In all, 74 publications were chosen for this systematic review.

In this PRISMA chart, we classify all the data records, including the total reference number and exclusion and inclusion criteria in our study.

The number of studies covered in this review is 8, as shown in the PRISMA figure above. The following is a list of the studies that were included in our systematic review. For each research, we reviewed the author, year of publication, country, title, sample size, as well as the study's purpose and methodology. Finally, we looked at the major results and references (Supplemental Table).

In this study, the authors analyzed data from 428 women across 8 included papers, with a total of 453 breast implants. Participants were aged 27 to 53 years, with an average age of 31. Breast implants were primarily used for cosmetic (94%) rather than reconstructive (6%) purposes. However, only 1 study specifically examined the distribution of women between aesthetic and reconstructive groups.

The reviewed studies largely lacked details on breast implant characteristics, such as type, size, surface, shape, material, and surgical placement techniques. Regarding microbiome data, 4 out of 8 studies used cell culture and pathology, whereas 1 employed a polymerase chain reaction assay. The most commonly detected bacterial species included *S. epidermidis*, *P. acnes*, *Streptococcus* spp., and *Staphylococci.* Other less frequently reported species included *Achromobacter xylosoxidans*, *Streptococcus viridans, Escherichia coli*, and *Bacillus cereus.*

**Figure 2.**
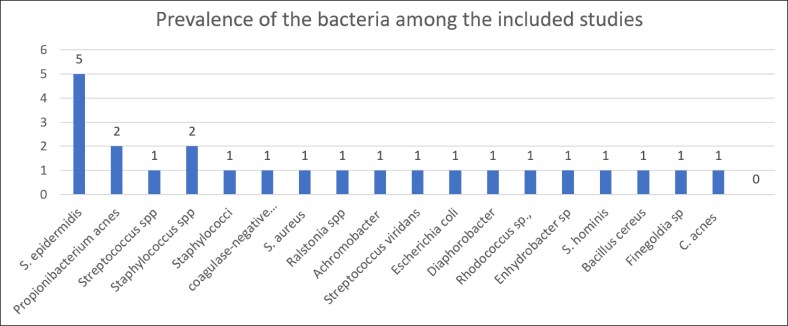
This figure illustrates bacterial prevalence across 8 studies, highlighting *Staphylococcus epidermidis* as the most frequent, *Propionibacterium acnes* and *Staphylococcus* spp. with moderate frequency, and other bacteria with mild occurrence.

Out of 428 patients, 106 had bacterial infections based on data from 8 studies. *Staphylococcus epidermidis* was the most common (81 cases), followed by *P. acnes* (10), *Bacillus* spp. (4), and *Staphylococcus aureus* (4). Additionally, *Haemolytic streptococci* and *Staphylococcus hominis* were found in 2 patients each, whereas *Achromobacter xylosoxidans*, *Escherichia coli*, and *Streptococcus viridans* were each identified in 1 patient (Figure 2).

Out of 453 analyzed breasts, 301 (66.44%) had CC. In only 1 study, the authors reported severity, with 12 cases of Grade 1, 16 of Grade 2, 10 of Grade 3, and 12 of Grade 4. No data on BIA-ALCL were reported.

## DISCUSSION

CC remains a significant complication following breast augmentation and reconstruction, affecting both patient outcomes and surgical success. This systematic review highlights the critical role of bacterial biofilms in the pathogenesis of CC, with *S. epidermidis*, *P. acnes*, and *Streptococcus* spp. being the most frequently isolated microorganisms from contracted breast capsules.^3,4^ These findings reinforce the hypothesis that bacterial contamination during implantation contributes to chronic inflammation and fibrosis around breast implants.^3,5^

Despite advancements in surgical techniques and implant materials, the incidence of CC remains high, with rates ranging between 5% and 74% depending on implant type and placement.^1,8^ Several factors contribute to its development, including implant type, surgical pocket selection, and postoperative infection control.^5,6^ The presence of bacterial biofilms has been implicated as a key driver of fibrosis, because microorganisms within biofilms evade host immune responses and persist on implant surfaces.^2,4^ A recent systematic review demonstrated that over 90% of *S. epidermidis* isolates from Baker grade III-IV capsules exhibit biofilm-forming capabilities, further supporting its role in contracture severity.^3^

Some studies suggest that surgical techniques and antiseptic protocols may influence bacterial contamination risk; however, detailed evaluation of prevention strategies was beyond the scope of this microbiota-focused review.^9-11^

The surface texture of breast implants has also been implicated in biofilm adhesion and contracture risk. Although textured implants have been associated with lower CC rates, they have been linked to an increased risk of *Breast Implant-Associated Anaplastic Large Cell Lymphoma (BIA-ALCL)*.^7,12^ Ralstonia species have been detected in some cases of BIA-ALCL; however, current evidence is insufficient to establish a direct causal relationship. These findings should be interpreted with caution, and further research is needed to clarify whether the presence of Ralstonia reflects contamination, opportunistic colonization, or a contributory factor.^3^ This underscores the need for further investigation into the long-term safety of textured implants and their association with chronic inflammation and lymphoproliferative disorders.

A critical gap in current research is the role of host immune responses in CC formation. Emerging evidence suggests that individual genetic predisposition and immunological variability may influence contracture severity and recurrence rates.^3,13^ The interplay between the host immune system and implant-associated biofilms remains poorly understood, highlighting the need for future studies incorporating immunological and microbiological data to develop targeted prevention strategies.

This review advances current understanding of CC by systematically consolidating evidence on the specific microbial species consistently detected across independent studies. Although previous reports have documented microbial presence in pathologic capsules, our synthesis uniquely highlights recurring patterns—particularly the predominance of *S. epidermidis* and *P. acnes*—across diverse patient cohorts and clinical settings. By mapping these findings against reported perioperative antisepsis protocols, this review identifies a potential protective association between rigorous antiseptic measures and reduced CC rates, warranting targeted future trials. Such synthesis can guide the design of standardized microbiological investigations and inform clinical strategies that directly address biofilm-associated implant complications.

### Limitations

Despite efforts to ensure a comprehensive analysis, this study has certain limitations. The exclusion of articles without CC data narrowed the scope of included studies. Additionally, only English-language publications were considered, potentially overlooking relevant findings in other languages. The authors of the study focused strictly on the association between breast microbiota and CC, specifically incorporating studies that examined both factors. Consequently, research outside this scope, unpublished data, or studies with ambiguous findings were excluded, which may have introduced selection bias.

Furthermore, the exclusion of studies with negative or inconclusive outcomes could impact the overall assessment, as such studies are less likely to be published. This publication bias may affect the generalizability of findings and limit a broader understanding of the etiopathogenesis of CC. Future research should incorporate a wider range of studies, including multilingual publications and those with diverse methodologies, to provide a more comprehensive analysis of risk factors and microbial influences on CC.

## CONCLUSIONS

This systematic review of 8 studies, involving 428 women and 453 breast implants, found that *S. epidermidis*, *P. acnes*, and *Streptococcus* spp. were the most commonly isolated microorganisms from contracted breast capsules. Across included studies, *S. epidermidis* was the predominant species, frequently associated with bacterial biofilm formation. The majority of microbiological assessments were performed using cell culture and pathology, with limited use of molecular techniques. Reporting of implant characteristics, surgical techniques, and severity grading was inconsistent, limiting direct comparisons between studies. These findings support an association between specific breast microbiota and CC.

## Supplemental Material

This article contains supplemental material located online at https://doi.org/10.1093/asjof/ojaf128.

## Supplementary Material

ojaf128_Supplementary_Data
