# Supplementary material for: Association Between Breast Microbiota and Capsular Contracture: A Systematic Review
Source: Aesthet Surg J Open Forum. 2025 Oct 7;7:ojaf128. doi: 10.1093/asjof/ojaf128 (PMC12611302; doi:10.1093/asjof/ojaf128)
Supplement: ojaf128_Supplementary_Data [file ojaf128_supplementary_data.docx]

| **Reference** | **Objective/ Purpose** | **Sample** | **Method** | **Key findings** |
| --- | --- | --- | --- | --- |
| 1/ Honghua et al, 2016  Title: Bacterial Biofilm Infection Detected in Breast Implant-Associated Anaplastic Large-Cell Lymphoma.  Country: United States of America  Reference link:  <https://journals.lww.com/plasreconsurg/Abstract/2016/06000/Bacterial_Biofilm_Infection_Detected_in_Breast.1.aspx> | To see if the bacterial biofilm is present in breast implant-associated Anaplastic large cell lymphoma (ALCL) and, if it is, to compare the bacterial microbiome to nontumor capsule samples from contractures breast implants. | 26 breast implants from 22 patients, 3 samples from anaplastic large cell lymphoma (ALCL) patients' contralateral normal breast capsules, and 62 non-tumour specimens. | Bacterial load, Bacterial Community Profiling, Scanning Electron Microscopy, and Fluorescent in Situ Hybridization were used to assess the presence of bacteria. | The presence of bacterial biofilm and a unique microbiome in breast implant-associated anaplastic large cell lymphoma (ALCL) samples suggests an infectious origin. |
| 2/ Bachour et al .2019  Title: PCR Characterization of Microbiota on Contracted and Non- Contracted Breast Capsules.  Country: Netherlands.  Reference link:  <https://link.springer.com/article/10.1007%2Fs00266-019-01383-9> | A very sensitive PCR test was used to identify the bacterial microbiota on normal and constricted breast capsules. | Females having reduction mammoplasty provided 50 breast capsules, as well as 10 glandular breast tissue and breast skin specimens. | IS-pro, a 16S-23S interspace region-based PCR test, was used to examine the samples. | The presence of microorganisms in normal and constricted capsules was the same. |
| 3/ J. Carvajal et al ,2018  Title: Back to Basics: Could the Preoperative Skin Antiseptic Agent Help Prevent Biofilm-Related Capsular Contracture?  Country: Colombia  Reference link:  <https://academic.oup.com/asj/article/39/8/848/5077082> | To compare the effects of two antiseptic skin preparations: povidone-iodine (PVP-I) and chlorhexidine gluconate (CHG) on capsular contracture proportions following primary breast augmentation using a periareolar technique. | The study assessed 63 individuals who had primary bra east augmentation using a periareolar technique in 2014. | Using PVP-I and CHG for surgical site preparation and comparing the results. PVP-I was utilised in 32 patients in the first six months, and CHG was used in 31 patients thereafter. | CHG was found to be more effective than PVP-I as a preoperative skin antiseptic for primary breast augmentation surgery in preventing biofilm-related Capsular contracture. |
| 4/ Chang et al, 2011  Title: Late hematogenous bacterial infections of breast implants: two case reports of unique bacterial infections  Country: United States of America  Reference link:  <https://journals.lww.com/annalsplasticsurgery/Abstract/2011/07000/Late_Hematogenous_Bacterial_Infections_of_Breast.6.aspx> | Cases of late infections of breast implants caused by hematogenous dissemination of bacterial infection from distant locations will be shown. | 2 patients | Case report studies | To avoid late breast infections and capsular contractures, systemic antibiotic treatment should be explored in breast implant patients who are at risk of bacterial inoculation and bacteremia. |
| 5/ Cohen et al, 2020  Title: Explanation in Tissue Expander and Direct-to-Implant Reconstruction with Acellular Dermal Matrix: How to Avoid Early Reconstructive Failures  Country: United States of America  Reference link:  <https://journals.lww.com/plasreconsurg/Abstract/2021/04000/Explantation_in_Tissue_Expander_and.3.aspx> | Explantation predictors in patients with acellular dermal matrix reconstructions will be investigated, as well as salvage strategies will be discussed. | Over the course of four years, 137 individuals received 234 unique breast reconstructions. | Retrospective reviews that have been approved. | In implant-based reconstruction with an acellular dermal matrix, skin necrosis is a severe risk factor for explantation. The reconstructive surgeon should consider early excision of any skin necrosis as soon as it is found. |
| 6/ Jonathan Cook et al, 2020  Title: Characterizing the Microbiome of the Contracted Breast Capsule Using Next Generation Sequencing.  Country: United States of America  Reference link:  <https://academic.oup.com/asj/article/41/4/440/5820223> | To describe the bacterial microbiome of the capsule using next-generation sequencing (NGS) in patients with Capsular contracture after cosmetic breast augmentatio | Following augmentation mammoplasty, 32 patients with Baker grade III or IV capsular contracture were studied. | MicroGenDX Laboratories (Lubbock, TX) received tissue specimens, intraoperative swabs of the breast capsule, and implant surfaces for NGS. | The immunological and inflammatory responses may be affected locally and systemically by dysbiosis of the breast microbiome, which might be the cause of Capsular contracture (CC), Anaplastic large cell lymphoma (ALCL), or breast implant-associated disease. |
| 7/ Crowe et al, 2021  Title: Microbial community compositions in breast implant biofilms associated with contracted capsules.  Country: Canada  Reference link:  <https://journals.plos.org/plosone/article?id=10.1371/journal.pone.0249261> | To evaluate the microbial community compositions of capsule biofilms. | The sample series consists of 17 cases in a series. | Using DNA isolated from breast implant capsule samples, amplicon sequencing of the 16S rRNA gene was performed. | More than 80% of capsules examined possessed amplifiable microbial 16S rRNA genes, which is equivalent to or greater than findings from earlier amplicon-based capsular contracture investigations (42 %, and 8 %). |
| 8/ Zhang et al, 2021  Title: Proper Skin Management in Breast Augmentation with a Peri areolar Incision Prevents Implant Contamination and Biofilm-Related Capsular Contracture.  Country: China  Reference link:  <https://link.springer.com/article/10.1007%2Fs00266-021-02205-7> | To suggest a skincare method for periareolar incisions to avoid implant contamination and biofilm-related capsular contracture. | A total of 129 patients were included in the study. | Randomized controlled trails. | Preoperative skincare helps maintain the surgical field clean and may even avoid implant infection and biofilm-related Capsular contracture. |

**Supplementary Table: List of the studies that were included in our systematic review.**
